# Factors affecting pitch discrimination performance in a cohort of extensively phenotyped healthy volunteers

**DOI:** 10.1038/s41598-017-16526-8

**Published:** 2017-11-28

**Authors:** Lauren M. Smith, Alex J. Bartholomew, Lauren E. Burnham, Barbara Tillmann, Elizabeth T. Cirulli

**Affiliations:** 1 University Program in Genetics and Genomics, Duke University, Durham, NC, 27708 USA; 2 Department of Molecular Genetics and Microbiology, Duke University School of Medicine, Durham, NC 27708 USA; 3 Lyon Neuroscience Research Center, Auditory Cognition and Psychoacoustics Team, CNRS-UMR 5292; INSERM, U1028, Lyon, F-69000 France; 4 University Lyon 1, Villeurbanne, F – 69000 France

## Abstract

Despite efforts to characterize the different aspects of musical abilities in humans, many elements of this complex area remain unknown. Musical abilities are known to be associated with factors like intelligence, training, and sex, but a comprehensive evaluation of the simultaneous impact of multiple factors has not yet been performed. Here, we assessed 918 healthy volunteers for pitch discrimination abilities—their ability to tell two tones close in pitch apart. We identified the minimal threshold that the participants could detect, and we found that better performance was associated with higher intelligence, East Asian ancestry, male sex, younger age, formal music training–especially before age 6–and English as the native language. All these factors remained significant when controlling for the others, with general intelligence, musical training, and male sex having the biggest impacts. We also performed a small GWAS and gene-based collapsing analysis, identifying no significant associations. Future genetic studies of musical abilities should involve large sample sizes and an unbiased genome-wide approach, with the factors highlighted here included as important covariates.

## Introduction

The ability to perceive differences in musical pitch can show great variation from person to person, with some having particularly good discrimination capacities (e.g.^1^). Beyond this variability, approximately 1–2% of the population has been estimated to have congenital amusia, a developmental disorder that affects pitch discrimination and memory as well as the perception of music^2^. Impaired pitch processing can also be caused by brain injury; for example, it occurs in some 50% of stroke patients (reviewed in^3^). In contrast, other individuals can perceive—and be annoyed by—even the slightest difference in pitch.

There have been numerous studies investigating the characteristics of human musical abilities. While some research has characterized aspects of musical traits as isolated from other brain-controlled traits, such as intelligence, hearing and speech (reviewed in^4^), there is also much evidence in support of shared neural networks between music and speech processing (for example,^5,6^). Previous studies have identified associations between intelligence and musical abilities, and there is even evidence that the association between musical ability and intelligence is due to common genetic factors affecting both traits^7–9^. Additionally, it is well known that there are associations between musical training and musical abilities, and there is evidence for differing musical abilities according to ethnicity and native language (for example, ref.^10,11^). Finally, previous studies have identified differences in musical pitch discrimination by sex, even after taking intelligence and musical training into account^12^. However, a full assessment of the various factors that could impact pitch discrimination that takes them all into account simultaneously in a large cohort has not yet been performed.

Multiple traits related to musicality have been shown to be heritable, including absolute pitch and rhythm discrimination^13^. The heritability of pitch discrimination ability has been estimated at 0.57–0.8^14,15^, but the specific genetic variants influencing this trait have yet to be identified. An analysis of pitch discrimination in over 200 Finns showed significant linkage to 4q22, and a family study in 148 Finns identified signatures of positive selection related to musical ability^15,16^. Thus far, the most statistically significant genetic association with a trait related to musicality comes from a study of pitch production accuracy in 1008 individuals from 73 Mongolian families. This study not only found linkage to 4q23 but also identified extremely significant associations (p = 8.0 × 10^−17^) with variants in or near the nearby gene *UGT8*, though it is not yet clear whether the causal variants driving this signal have been identified^17^.

Here, we improve knowledge about pitch discrimination by comprehensively studying its correlates in a population of 918 healthy volunteers. We also perform a small genome-wide association study and gene-based collapsing analysis of this trait. We encourage further research into the genetics of musical traits because a better understanding of the genetic variation influencing normal differences in auditory perception would be beneficial for research into patients with amusia and disorders related to auditory perception; examples include the language disorders of aprosodia and aphasia, which can be impacted by impaired processing of pitch information^18^.

## Materials and Methods

All methods were performed in accordance with the relevant guidelines and regulations.

### Participants

The Duke University Institutional Review Board approved all procedures, which were performed in accordance with relevant guidelines and regulations, and participants provided written, informed consent (IRB#: Pro00006828). We assessed 918 participants, ranging in age from 18 to 82 years, for pitch discrimination as part of a larger battery in the Duke Genetics of Cognition and Other Normal Variation study^19–21^. A description of the participants can be seen in Table 1, and all collected data can be found in Table S1. Many of the participants were university students, and some were international students.

**Table 1:** Participant Demographics.

Variable	Mean (SD) or Count (%)
Age in years	25.4 (9.8)
Ancestry	
European	422 (46.0%)
African	139 (15.1%)
East Asian	135 (14.7%)
South Asian	74 (8.1%)
Hispanic	69 (7.5%)
Other	79 (8.6%)
Sex	
Male	339 (36.9%)
Female	579 (63.1%)
Education	
Years of education	15.2 (2.0)
Current student	643 (70.0%)
CIRENS	0.45 (1.2)
EPQ-BV	
Extraversion	38.3 (8.6)
Neuroticism	27.0 (8.0)
Musical training	
Formal musical training	492 (53.6%)
Before the age of six	154 (16.8%)
Years musical training (if any; n = 461)	8.9 (5.6)
Synesthesia	20 (2.2%)
Misophonia (n = 500)	11 (2.2%)
Absolute pitch	
Self-report absolute pitch	58 (6.3%)
Tested for absolute pitch	21 (2.3%; 36.2% of 58)
Confirmed to have absolute pitch	7 (0.8%; 33.3% of 21)

Standard deviation (SD), Circadian Energy Scale (CIRENS), Eysenck Personality Questionnaire – Brief Version (EPQ-BV). Note that the sample size for misophonia is smaller as only 500 participants were queried, and years of musical training is only available for 461 of 492 participants with musical training due to some ambiguous responses (see Methods). For absolute pitch, only those who reported having absolute pitch were tested; many who reported having absolute pitch were not tested due to failure to follow up.

### Questionnaire

Prior to psychometric testing, the participants completed an extensive survey that queried demographics, medical history, and several standardized scales as follows and shown in Table 2 ^22^.

**Table 2:** Associations with pitch discrimination.

Variable	Univariate p	Univariate beta	Univariate r^2^	Multivariate p	Multivariate beta
Intelligence	**<0.001**	**−0.07261**	**0.2261**	**<0.001**	**−0.0498**
Ancestry					
African	**<0.001**	**0.2045**	**0.0611**	0.015	0.0619
East Asian	**<0.001**	**−0.1677**	**0.0358**	**<0.001**	**−0.0976**
South Asian	**0.005**	**0.1005**	**0.0085**	0.012	0.0852
Hispanic	NS	NS	NS	0.468	−0.0240
Other/mixed	NS	NS	NS	0.447	−0.0229
Male	**0.003**	**−0.0600**	**0.0095**	**<0.001**	**−0.1037**
Age	**<0.001**	**0.0081**	**0.0717**	**0.001**	**0.0032**
Education				NS	NS
Years of education	NS	NS	NS		
Current student	<**0.001**	**−0.1316**	**0.0413**	NS	NS
CIRENS	**0.002**	**−0.0249**	**0.0101**	NS	NS
Musical training					
Formal training	**<0.001**	**−0.2119**	**0.1270**	**<0.001**	**−0.1044**
Before age of six	<**0.001**	**−0.2127**	**0.0718**	<**0.001**	**−0.0847**
EPQ-BV	NS	NS	NS	NS	NS
Extraversion					
Neuroticism					
English first language	**0.001**	**−0.09162**	**0.0127**	**0.004**	**−0.0751**
Synesthesia	NS	NS	NS	NS	NS
Misophonia (n = 500)	NS	NS	NS	NS	NS
Absolute pitch	**<0.001**	**−0.4659**	0.0187	NS	NS

Bolded *p* values are < 0.01. NS indicates p > 0.05. Lower pitch discrimination values reflect better scores. Note that the sample size for misophonia was smaller as only 500 participants were queried.

#### Circadian rhythms

The Circadian Energy Scale (CIRENS) is a two-question chronotype measure based on self-report energy levels throughout the day: once at night and once in the morning. Energy levels are described on a Likert scale: [very low (1), low (2), moderate (3), high (4), or very high (5)]. The difference between the evening score and morning score determines the overall chronotype score, ranging from −4 (most marked morning preference) to +4 (most marked evening preference)^23^. Scores of −2, −3 and −4 are considered morning type, while scores of 2, 3 and 4 are considered evening type, and scores of −1, 0 and 1 are considered neither.

#### Extraversion and neuroticism

The Eysenck Personality Questionnaire, Brief Version was employed and includes two scales of 12 questions for both extraversion and neuroticism^24^. Each question asked about personal traits, like “Are you a talkative person?” and “Are your feelings hurt easily?” and had multiple choice answers, rated as Not at all = 1, Slightly = 2, Moderately = 3, Very Much = 4, and Extremely = 5. The maximum possible score for each subscale is 60, with higher scores meaning greater levels of extraversion or neuroticism.

#### Musical training

All participants indicated whether or not they had ever received formal musical training and whether or not that training had commenced before the age of six years old, as this has been shown to be a critical time period in the development of advanced musical skills like absolute pitch^25^. Participants were also asked at what ages they received musical training, although we set to missing the answers from 31 participants who gave ambiguous answers, like “age 12,” which did not make clear whether they had just received one year of training or had trained every year since age 12.

#### Synesthesia, misophonia, and absolute pitch

Participants were asked the following questions: “(1) Do you have any type of synesthesia? Synesthesia is a perceptual phenomenon where sensations are mixed (for example, numbers or letters are represented by colors or shapes, or days of the week have different physical locations); ” (2) “Do you have misophonia? Misophonia is a neurological disorder where specific sounds (usually those of a repetitive nature such as breathing, or slurping food) cause anger, hatred, disgust, or impulsive aggression;” and (3) “Do you have perfect or absolute pitch? Perfect or absolute pitch is the ability of a person to hear a note and be able to identify it immediately, for example, ‘that’s a C#.” Fifty-eight participants responded affirmatively regarding absolute pitch; twenty-one of them were then tested for their ability to correctly name notes via testable.org/t/281cab17 with a 25 pure tone paradigm based on a previous study^26^, with 1 point given for each correct response and 0.75 points given for each response that was only off by a semitone. Participants were considered to have absolute pitch if they scored at least a 15.

### Cognitive test

All participants took a brief battery of eleven standardized, well-known cognitive tests assessing diverse areas of cognition represented in Table 3 ^19^. Principal component analysis was performed on the individual test scores to determine an overall measure of performance^19^. The first principal component (PC1) explained 41.5% of the total variation in test scores and received approximately equal loadings from all tests (Table 3). It was therefore taken as a measure of overall cognitive performance on the battery and can be considered a proxy for general intelligence.

**Table 3:** Cognitive correlates with pitch discrimination.

Test	PCA loading for general intelligence	Cognitive Area	r with pitch discrimination	r with duration discrimination^21^
Stroop Color-Word^50^	0.33	Attention, Executive Control	−0.43	−0.28
TrailsB^51^	−0.36	Attention, Processing Speed, Executive Control	0.37	0.26
Symbol Search^52^	0.34	Processing Speed, Executive Control	−0.33	−0.23
Digit Symbol^52^	0.29	Processing Speed, Working Memory, Executive Control	−0.31	−0.16
TrailsA^51^	−0.30	Attention, Processing Speed	0.29	0.15
Immediate Story Recall^53^	0.32	Verbal Episodic Memory	−0.28	−0.16
Delayed Story Recall^53^	0.33	Verbal Episodic Memory	−0.28	−0.17
Animals^54^	0.32	Semantic Fluency	−0.26	−0.15
COWA^55^	0.26	Verbal Fluency, Executive Control	−0.24	−0.19
Digit Span Backward^52^	0.25	Working Memory	−0.23	−0.16
Digit Span Forward^52^	0.19	Working Memory	−0.21	−0.11

Correlations between subtests of the cognitive battery and timing performance are presented as Pearson’s r. Lower pitch discrimination scores indicate better performance, and higher cognitive test scores (except for Trails) indicate better performance. All *p* < 0.001.

### Pitch and Duration Discrimination

Participants completed an auditory pitch discrimination task using a classic adaptive procedure implemented by the MLP MATLAB toolbox^27^. In each trial, randomized 250 msec pure tones with raised cosine onset and offset gates of 10-msec were presented in a three-alternate forced choice task at 75 dBA. Participants judged which of the three tones, two of which were the same, was highest in pitch, and immediate feedback was given in the form of “correct” or “incorrect”. A standard 1 kHz pure tone was used as the baseline and the pitch of the remaining tones was determined in real time based on participant responses and presented with 500-msec silent intervals. The maximum likelihood procedure tracked the 79% threshold of the participant’s psychometric function, and an independent threshold was independently generated three times using 30 trials. No training trials were performed. Participants were required to pass a hearing test with no more than 10 dBA of hearing loss to be eligible^27^.

Duration discrimination was also assessed using the MLP MATLAB toolbox as previously described^21^; the paradigm was the same as that for pitch discrimination, except that the frequency was held at 1 kHz, and the duration was altered between trials, with 250 msec as the baseline and the participant judging which of the three tones, two of which were the same, was longest. For both pitch discrimination and duration discrimination, the thresholds were log transformed, and then the median of three independent thresholds output by the maximum likelihood procedure per person were taken as their phenotype. While the MLP can be sensitive to errors that occur in the first few trials, our use of the median from three independent threshold estimations mitigated potential problems from this issue; in fact, we found that the median log transformed threshold was well correlated with the best log transformed threshold for each person (r^2^ = 0.89, p < 0.001), with no obvious outliers. To approximate a normal distribution, the median scores for each person were then Box Cox transformed (((threshold^L)−1)/L; pitch L = −0.1208564; duration L = −0.3825963)^28^.

### Repeat Sessions

To evaluate the reliability of our tasks, 56 participants completed the pitch and duration discrimination tasks twice. The mean amount of time between testing sessions was 71 days (SD = 30) (Table S2).

### Data Analyses

Non-genetic statistical analyses were performed using STATA^29^. Stepwise forward linear regression analyses with a cutoff for inclusion of *p* < 0.01 were performed, with the pitch discrimination as the outcome and all variables listed in Table 2 as covariates. Variables that were significant in the stepwise model were used as covariates in subsequent genetic analyses. The residuals of the linear analysis approximated a normal distribution.

### Genetic Analyses

Power calculations were performed using GWASpower/QT (available at http://igm.cumc.columbia.edu). A genome-wide association study (GWAS) was performed on 179 participants of European ethnicity who had Illumina Humanexome chip data available. Of these 179, most were also genotyped with the Infinium HumanCore GWAS chip (n = 139), and others were genotyped with either the Human610-Quad BeadChip (n = 10) or HumanHap550 (n = 18). Twelve of these 179 samples did not have additional GWAS genotypes. Imputation was performed on each of the chip-specific subgroups with the Michigan Imputation Server using the default settings and the Haplotype Reference Consortium dataset, and the subsequent data were then merged, with variants with r^2^ < 0.3 excluded^30,31^.

Our single variant analysis restricted to variants genotyped in at least 40% of these participants. A linear regression analysis was performed in plink^32^. Two EIGENSTRAT axes, PC1, sex, age and musical training were used as covariates in each analysis. A total of 10,090,562 variants were analyzed in this GWAS. We used the standard p-value cutoff of 5 × 10^−8^ to correct for multiple tests^33^.

To assess the effects of the low frequency variants genotyped with the exome chip, we used a gene-based collapsing analysis as previously described^34^. Briefly, we summarized for each participant whether there existed a ‘qualifying’ variant in each gene, where qualifying was defined as an exonic variant with MAF < 0.01. Multivariate linear regression analysis was then performed with two EIGENSTRAT axes and PC1 as covariates. This allows the identification of genes where qualifying variants are enriched in individuals toward one extreme or the other of each trait.

We also performed targeted analyses of our data that focused on candidate regions implicated in previous studies of traits related to musicality (coordinates according to GRCh37/hg19): chromosome 4 88.0–100.8 MB (4q22)^15^; chromosome 498–120 MB (~4q23–4q26)^17^; and chromosome 3127.2–129.2 MB (2 MB around rs9854612) and chromosome 429.6–31.6 MB (2 MB around rs13146789)^35^.

### Data availability

All non-genetic data used in this study are present in Table S1 and S2. The genetic data can be found in dbGaP study phs001406.

## Results

### Distribution and test-retest reliability

After Box Cox transformation, the cohort presented an approximately normal distribution of pitch discrimination ability. The median threshold for telling two pitches apart was 8.41 Hz (14.50 cents); the best 5% of participants could distinguish a difference of 3.58 Hz (6.19 cents), and the worst 5% could only distinguish a difference of 45.71 Hz (77.38 cents). The tests showed high reliability: for the 56 participants who took the measurements twice on separate days, the correlation coefficient between pitch discrimination performance at the first and second session was 0.87 (p < 0.001), while the correlation between the two sessions for duration discrimination was 0.74 (p < 0.001).

### Association with covariates

Using stepwise forward linear regression analysis, the results of which incorporate the effects of multiple covariates simultaneously, we found significant associations between better pitch discrimination performance and higher intelligence, East Asian ancestry, male sex, younger age, formal music training–especially before age 6–and English as the native language. Details can be seen in Table 2. No significant associations were seen for personality traits, circadian preference, misophonia or synesthesia. Students appeared to have significantly better pitch discrimination performance according to a univariate regression, but multivariate regression showed that this effect was due to the association between education and age. Similarly, morningness-eveningness preference was correlated with pitch discrimination in the univariate analysis but was explained by other variables, primarily age, in the multivariate analysis. Possessing absolute pitch was associated with better pitch discrimination, but the low sample size (only 7 participants with confirmed absolute pitch) led to this variable not passing the p < 0.01 inclusion threshold for the multivariate model. Altogether, multivariate regression showed that a model including all statistically significant covariates shown in Table 2 could explain 34.5% of the variation in pitch discrimination performance.

We used the responses to our binary questions of whether the participant had received musical training and whether they had been taught prior to the age of 6 as opposed to using the quantitative measure of the total number of years of musical training due to some missing data from ambiguous responses. However, we found that after excluding the 31 participants with missing data, years of musical training—as a quantitative covariate, including 0 for those with no training—was a slightly better predictor of pitch discrimination performance (p < 0.001, r^2^ = 0.150) than was the combination of these binary variables (p < 0.001, r^2^ = 0.144).

### Cognitive correlates

Each of the eleven cognitive measures of our cognitive battery were strongly (*p* < 0.001) associated with pitch discrimination performance (Table 3). Interestingly, the highest correlations seen between pitch discrimination and specific cognitive areas were for executive control and attention. This result is similar to our previous report on the cognitive performance areas associated with duration discrimination, where overall cognitive performance showed the best association with discrimination threshold, and the tests assessing executive function were the most important contributors to that association. In fact, none of the cognitive tests were significantly (p < 0.01) associated with pitch discrimination performance after accounting for overall cognitive performance, except for performance on the Stroop Color-Word, better performance on which remained strongly associated with better pitch discrimination (p < 0.001) even after accounting for general intelligence.

We found that performance on the duration discrimination task was also significantly associated with pitch discrimination, explaining 15.8% of the variance in this trait (beta = 1.5089; p < 0.001). Multivariate analysis showed that the association with duration discrimination could not be fully explained by any of the significant covariates shown in Table 2, including general intelligence. The results showed that duration discrimination and general intelligence were both separately and strongly associated with pitch discrimination (duration beta = 0.9980, p < 0.001; intelligence beta = −0.0545, p < 0.001).

### Genetic associations

After correcting for multiple tests, we identified no variants or genes with statistically significant associations with pitch discrimination. Our small sample size of 179 participants with genetic data left us very underpowered; the genome-wide association study had 80% power to identify a common variant explaining at least 18% of the variation in this trait, and our gene-based collapsing analysis of low-frequency coding variants had 80% power to identify associations explaining at least 14% of the variation. This remained true when focusing on regions on chromosomes 3 and 4 that had previously been found to be linked to pitch discrimination^15,17,35^. We also did not find an association between pitch discrimination and rs4148254, which had not previously been specifically investigated with regard to pitch discrimination but does currently have the most statistically significant genetic association reported for a musical trait, in this case pitch production accuracy^17^. We were powered to identify this variant as significant if it explained at least 3.5% of the variation in pitch discrimination, but it is worth mentioning that we only had 19 variant carriers out of 179. The previous study was performed in East Asians, where the frequency of this variant is higher than in the European American participants we were able to include in this analysis.

## Discussion

Here, we present a study on the associations between various genetic and non-genetic factors and auditory pitch discrimination in a large cohort of healthy volunteers. Our findings reveal comprehensive information about the variables that influence pitch discrimination. We confirm the importance of variables previously reported to associate with pitch discrimination, and our large sample size and diverse population allow us to rank the importance of each of these variables to performance. Consistent with previous studies, we find that general intelligence plays the largest role in pitch discrimination, explaining 23% of the variation^7^. Because general intelligence was also associated with the related duration discrimination task^21^ (p < 0.001; r^2^ = 12%), it seems likely that general intelligence influences performance on the testing paradigm that is employed for both tasks, and that the true influence of general intelligence on the ability to discern different pitches is somewhat less than is suggested here. This observation also motivates future studies that should instead use indirect investigation methods to measure pitch discrimination capacities more specifically; it has been shown in various domains that implicit measures reveal increased processing capacities compared to explicit measures (e.g.,^36,37^). Furthermore, previous studies have investigated the link between cognitive performance and auditory skills and have supported a model where specific aspects of auditory perception and general intelligence work together to determine an individual’s performance on a particular auditory task^8^. This relationship is in line with a bottom-up model, where there are specific neural and cellular properties that affect both cognitive and auditory tasks, as opposed to a top-down model, where general intelligence directly influences auditory tasks and there is no separate auditory skillset^38^.

Duration discrimination performance on its own explains 16% of the variation in pitch discrimination performance. Multivariate regression shows that, together, duration discrimination performance and overall cognitive performance can explain 29% of the variation in pitch discrimination performance, with both measures having strong impacts (p < 0.001) on pitch discrimination even when controlling for the other test (i.e., duration discrimination or cognitive performance). In comparison, cognitive performance only explains 12% of the variation in duration discrimination, and adding pitch discrimination in a multivariate regression only brings the total amount explained up to 19%. Despite the greater contribution of cognitive performance to pitch discrimination than to duration discrimination, our results showed that the associations between these two traits and cognitive performance were due to impacts from similar cognitive domains, with the executive function and attention components of general intelligence playing the biggest roles^21^. Interestingly, our multivariate regression showed that Stroop Color-Word, which assesses executive function, was the only component of our cognitive battery to have an association with pitch discrimination performance (and with duration discrimination) that remained significant (p < 0.001) after controlling for the association with overall cognitive performance. It should be noted that our participants were healthy volunteers from the normal range of intelligence in the general population, and so the observed associations between cognitive performance and auditory tasks do not imply that those with amusia have cognitive deficits. Additionally, our results support previous findings that poor pitch discrimination or duration discrimination are not simply symptoms of general poor short term memory^39^. In fact, those in the top 10% or bottom 10% of pitch discrimination or duration discrimination performance showed essentially the same range of scores on the memory-assessing story recall and digit span tests as did the rest of our population (Figure S1). Given the known association between congenital amusia and impaired short-term memory for pitch, future studies should investigate more specifically the link between pitch discrimination and short-term pitch memory within the normal range analyzed here^40^.

**Figure 1:**
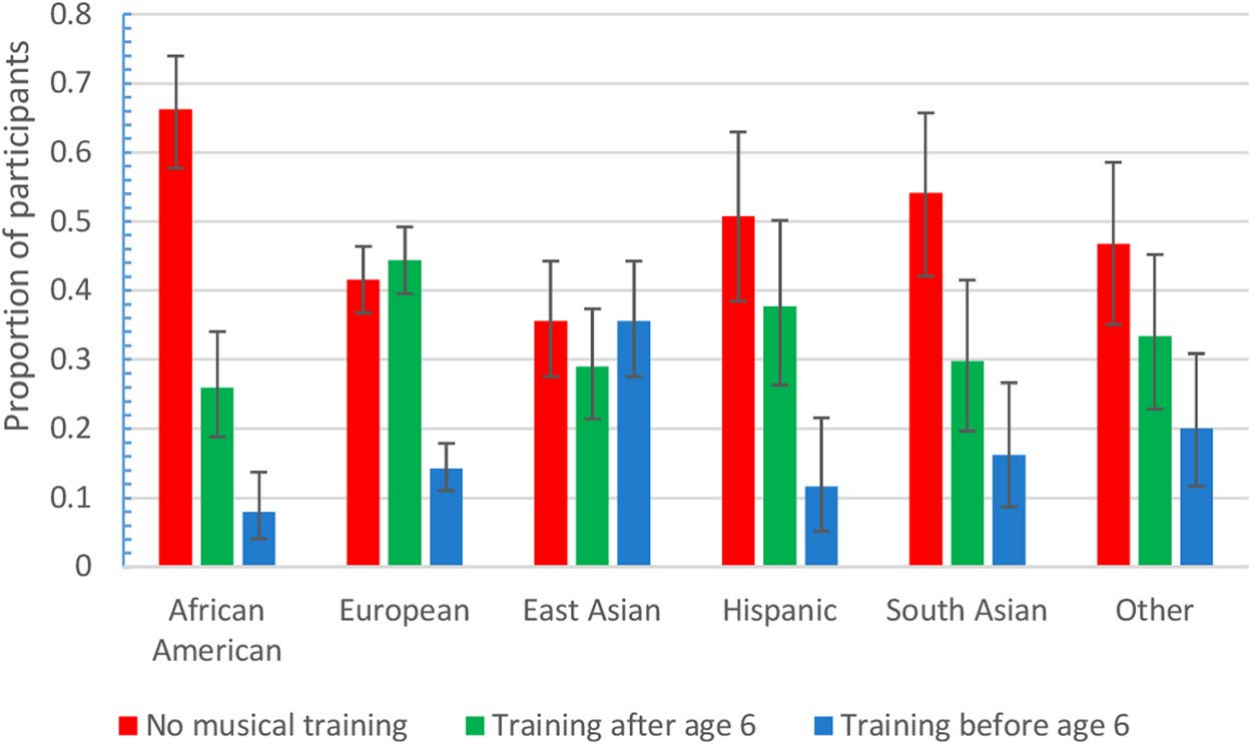
Proportion of participants of each ancestry type who reported receiving musical training. The error bars show the 95% confidence intervals.

The next most important factor in pitch discrimination was music training. The median pitch discrimination threshold for participants with formal music training was 7.45 Hz (12.85 cents; n = 492) as compared to 11.33 Hz (19.50 cents; n = 426; p < 0.001 (Table 2)) for those without formal music training, and the median for those with training before the age of six was even lower, at 6.48 Hz (11.18 cents; n = 154 p < 0.001 (Table 2)). The effect of musical training was not as large in our study as has been found in some previous studies (Figure S2), which may be expected because we were not able to separate out professional musicians from those with more modest levels of training^41^. However, most of our participants did indicate the total number of years of musical training they had received, and we found this quantitative variable to predict pitch discrimination performance only slightly better than did more general questions about whether they had had any musical training and had been trained before the age of six. Furthermore, when restricting to participants with musical training, the total number of years of training was only able to explain 7.2% of the variation in pitch discrimination performance (n = 461; linear regression p < 0.001). In contrast, there was no significant correlation between duration discrimination performance and number of years of training for participants who reported having had musical training. We also did not collect information about the type of music training or comprehensive information about the number of years of musical training, only whether participants reported ever having had formal music training and at what ages this training occurred. Because methods of musical training can vary by culture and may affect the development of musical abilities, cultural differences may influence the abilities measured here. We did find that the incidence of formal musical training varied greatly by ethnicity (Fig. 1).

Even after accounting for musical training, cognitive performance, and age, East Asian ancestry was strongly associated with better pitch discrimination performance; South Asian ancestry, which largely corresponded to Indian ancestry, trended toward better performance as well (Figure S3). While our results are in accordance with previous studies indicating that those of Asian ancestry have better pitch discrimination and a higher incidence of absolute pitch, it is not yet clear whether that difference is due primarily to genetic or cultural differences, including different methods of musical training^42–45^. While there has been some speculation that tonal languages aid in the development of musical abilities, we, consistent with previous studies^42^, found that among our participants who did not have English as their native language, those of Chinese ethnicity (tonal language, n = 36) performed no better than did our Korean ethnicity participants (non-tonal language, n = 9). It should be noted that we did not actually query the native language of the participants, so these analyses are based on self-reported ethnicity and self-reports of whether English was the native language. We also found that those who reported that English was their native language had a median pitch discrimination threshold of 8.42 Hz (14.52 cents) as compared to 10.34 Hz (17.81 cents) for those with a different native language (p = 0.001; p = 0.004 in multivariate analysis accounting for ethnicity, intelligence, etc. (see Table 2)). This association was seen across multiple ethnicities but had the strongest effect in non-native English speakers of South Asian ancestry, whose median pitch discrimination threshold was 14.25 Hz (24.50 cents) (n = 43). It appears that the improved performance of native English speakers is specific to pitch discrimination, as opposed to being due to the testing paradigm, as we found that duration discrimination was not affected by native language. Our result, which is generalizable across most non-native English speakers, is in contrast to previous work suggesting that tonal language speakers—specifically—have worse pitch discrimination^46^.

Finally, we found that males had significantly better pitch discrimination performance than did females, consistent with previous studies^12,47^. However, sex explained less than 1% of the variation in pitch discrimination performance, emphasizing that this is a significant but very imprecise predictor of performance.

Our study provides the first comprehensive assessment of diverse factors influencing auditory pitch discrimination in a large cohort of healthy volunteers. Our modest genetic analysis was not powered for new discoveries, but it also did not extend to pitch discrimination a previous association seen between pitch production accuracy and variation near the gene *UGT8* ^17^. This result is perhaps unsurprising as prior studies have provided evidence that singing abilities are largely unrelated to pitch discrimination^48,49^. Nonetheless, the proximity of the 4q23 locus implicated in the *UGT8* pitch production accuracy study to the 4q22 locus implicated in a previous study of different types of musical perception, especially auditory structuring ability, provided a tantalizing possibility for a genetic connection that could explain at least some combined portion of the variation in these traits^15,17^. These two prior studies have partial overlap in their linkage peaks, which could in theory reflect common genetic variation underlying different aspects of musicality. Unfortunately, our study does not provide new evidence to cement this link. We were also were unable to shed light on the causal variants that underlie other linkage regions found in previous studies of musical abilities^15,17,35^. While our genetic sample size was sufficient to provide 80% power to detect an association with the *UGT8* variant rs4148254 if it explained at least 3.5% of the variation in pitch discrimination in our sample, we were underpowered for novel genetic discovery throughout these linkage peaks. Future genetic analyses of this trait must have a large enough sample size to be well powered and take a genome-wide, unbiased approach for discovery.

## Electronic supplementary material

Supplemental Figures S1-S3

Supplemental Tables S1-S2

## References

[1] Foxton JM, Weisz N, Bauchet-Lecaignard F, Delpuech C, Bertrand O (2009). The neural bases underlying pitch processing difficulties. Neuroimage.

[2] Peretz, I. & Vuvan, D. T. Prevalence of congenital amusia. *Eur J Hum Genet* (2017). 10.1038/ejhg.2017.15 PMC5437896 28224991

[3] Sihvonen AJ (2016). Neural Basis of Acquired Amusia and Its Recovery after Stroke. J Neurosci.

[4] Pearce JM (2005). Selected observations on amusia. Eur Neurol.

[5] Groussard M (2010). Musical and verbal semantic memory: two distinct neural networks?. Neuroimage.

[6] Abrams DA (2011). Decoding temporal structure in music and speech relies on shared brain resources but elicits different fine-scale spatial patterns. Cereb Cortex.

[7] Mosing MA, Pedersen NL, Madison G, Ullen F (2014). Genetic pleiotropy explains associations between musical auditory discrimination and intelligence. PLoS One.

[8] Kidd GR, Watson CS, Gygi B (2007). Individual differences in auditory abilities. J Acoust Soc Am.

[9] Grassi M, Borella E (2013). The role of auditory abilities in basic mechanisms of cognition in older adults. Front Aging Neurosci.

[10] Pfordresher PQ, Brown S (2009). Enhanced production and perception of musical pitch in tone language speakers. Atten Percept Psychophys.

[11] Wong PC (2012). Effects of culture on musical pitch perception. PLoS One.

[12] Rammsayer TH, Troche SJ (2012). On sex-related differences in auditory and visual sensory functioning. Arch Sex Behav.

[13] Tan YT, McPherson GE, Peretz I, Berkovic SF, Wilson SJ (2014). The genetic basis of music ability. Front Psychol.

[14] Drayna D, Manichaikul A, de Lange M, Snieder H, Spector T (2001). Genetic correlates of musical pitch recognition in humans. Science.

[15] Pulli K (2008). Genome-wide linkage scan for loci of musical aptitude in Finnish families: evidence for a major locus at 4q22. J Med Genet.

[16] Liu X (2016). Detecting signatures of positive selection associated with musical aptitude in the human genome. Sci Rep.

[17] Park H (2012). Comprehensive genomic analyses associate UGT8 variants with musical ability in a Mongolian population. J Med Genet.

[18] Ross ED (1981). The aprosodias. Functional-anatomic organization of the affective components of language in the right hemisphere. Arch Neurol.

[19] Cirulli ET (2010). Common genetic variation and performance on standardized cognitive tests. European Journal of Human Genetics.

[20] Cirulli ET (2011). Contribution of pastimes and testing strategies to the performance of healthy volunteers on cognitive tests. The Clinical Neuropsychologist.

[21] Bartholomew AJ, Meck WH, Cirulli ET (2015). Analysis of Genetic and Non-Genetic Factors Influencing Timing and Time Perception. PLoS One.

[22] Harris PA (2009). Research electronic data capture (REDCap)—a metadata-driven methodology and workflow process for providing translational research informatics support. Journal of biomedical informatics.

[23] Ottoni GL, Antoniolli E, Lara DR (2011). The Circadian Energy Scale (CIRENS): two simple questions for a reliable chronotype measurement based on energy. Chronobiology International.

[24] Sato T (2005). The Eysenck personality questionnaire brief version: Factor structure and reliability. The Journal of Psychology: Interdisciplinary and Applied.

[25] Baharloo S, Johnston PA, Service SK, Gitschier J, Freimer NB (1998). Absolute pitch: An approach for identification of genetic and nongenetic components. The American Journal of Human Genetics.

[26] Baharloo S, Johnston PA, Service SK, Gitschier J, Freimer NB (1998). Absolute pitch: an approach for identification of genetic and nongenetic components. Am J Hum Genet.

[27] Grassi M, Soranzo A (2009). MLP: A MATLAB toolbox for rapid and reliable auditory threshold estimation. Behavior Research Methods.

[28] Box, G. E. P. & Cox, D. R. An analysis of transformations. *Journal of the Royal Statistical Society, Series B* 211–252 (1964).

[29] StataCorp. Stata Statistical Software. In *Stata Statistical Software* Vol. Release 13 (StataCorp LP, College Station, TX, 2013).

[30] Das S (2016). Next-generation genotype imputation service and methods. Nat Genet.

[31] McCarthy S (2016). A reference panel of 64,976 haplotypes for genotype imputation. Nat Genet.

[32] Purcell S (2007). PLINK: a tool set for whole-genome association and population-based linkage analyses. The American Journal of Human Genetics.

[33] McCarthy MI (2008). Genome-wide association studies for complex traits: consensus, uncertainty and challenges. Nat Rev Genet.

[34] Cirulli, E.T. *et al*. Exome sequencing in amyotrophic lateral sclerosis identifies risk genes and pathways. *Science*, aaa3650 (2015). 10.1126/science.aaa3650 PMC4437632 25700176

[35] Oikkonen J (2015). A genome-wide linkage and association study of musical aptitude identifies loci containing genes related to inner ear development and neurocognitive functions. Mol Psychiatry.

[36] Zendel BR, Lagrois ME, Robitaille N, Peretz I (2015). Attending to pitch information inhibits processing of pitch information: the curious case of amusia. J Neurosci.

[37] Tillmann B, Peretz I, Bigand E, Gosselin N (2007). Harmonic priming in an amusic patient: the power of implicit tasks. Cogn Neuropsychol.

[38] Ullén, F., Söderlund, T., Kääriä, L. & Madison, G. Bottom–up mechanisms are involved in the relation between accuracy in timing tasks and intelligence — Further evidence using manipulations of state motivation. *Intelligence*, 100–106 (2012).

[39] Williamson VJ, Stewart L (2010). Memory for pitch in congenital amusia: beyond a fine-grained pitch discrimination problem. Memory.

[40] Tillmann B, Leveque Y, Fornoni L, Albouy P, Caclin A (2016). Impaired short-term memory for pitch in congenital amusia. Brain Res.

[41] Micheyl, C., Delhommeau, K., Perrot, X. & Oxenham, A. Influence of musical and psychoacoustical training on pitch discrimination. *Hearing Research*, 36–47 (2006). 10.1016/j.heares.2006.05.004 16839723

[42] Gregersen, P. K., Taylor, K. E. & Li, W. Reply to Henthorn and Deutsch: Ethnicity versus early environment: Comment on ‘Early Childhood Music Education and Predisposition to Absolute Pitch: Teasing Apart Genes and Environment’ by Peter K. Gregersen, Elena Kowalsky, Nina Kohn, and Elizabeth West Marvin [2000] 10.1002/ajmg.a.31595/epdf) *Am J Med Genet* **143A**, 104–105 (2007). 10.1002/ajmg.a.31596 17163519

[43] Gregersen PK, Kowalsky E, Kohn N, Marvin EW (2001). Early childhood music education and predisposition to absolute pitch: teasing apart genes and environment. Am J Med Genet.

[44] Hove MJ, Sutherland ME, Krumhansl CL (2010). Ethnicity effects in relative pitch. Psychon Bull Rev.

[45] Henthorn, T. & Deutsch, D. Ethnicity versus early environment: comment on ‘Early childhood music education and predisposition to absolute pitch: teasing apart genes and environment’ by Peter K. Gregersen, Elena Kowalsky, Nina Kohn, and Elizabeth West Marvin [2000]. *Am J Med Genet A* **143A**, 102–3; author reply 104-5 (2007). 10.1002/ajmg.a.31596 17163519

[46] Peretz I, Nguyen S, Cummings S (2011). Tone language fluency impairs pitch discrimination. Front Psychol.

[47] Mosing MA, Madison G, Pedersen NL, Kuja-Halkola R, Ullen F (2014). Practice does not make perfect: no causal effect of music practice on music ability. Psychol Sci.

[48] Bradshaw E, McHenry MA (2005). Pitch discrimination and pitch matching abilities of adults who sing inaccurately. J Voice.

[49] Pfordresher PQ, Brown S (2007). Poor-Pitch Singing in the Absence of “Tone Deafness”. Music Perception: An Interdisciplinary Journal.

[50] Golden CJ (1975). The measurement of creativity by the Stroop Color and Word Test. Journal of Personality Assessment.

[51] Battery, A. I. T. Manual of directions and scoring. *Washington, DC: War Department, Adjutant General’s Office* (1944).

[52] Wechsler, D. & Scale, W. A. I. WAIS-III. *WMS-III technical manual* (1997).

[53] Green, P. Story recall test. *Edmonton (CA): Green’s Publishing* (2005).

[54] Rosen WG (1980). Verbal fluency in aging and dementia. Journal of Clinical and Experimental Neuropsychology.

[55] Benton, A., Hamsher, K. & Sivan, A. Multilingual Aphasia Examination. Iowa City, IA: AJA Associates. (Inc, 1989).

