# Supplementary material for: Factors affecting pitch discrimination performance in a cohort of extensively phenotyped healthy volunteers
Source: Sci Rep. 2017 Nov 28;7:16480. doi: 10.1038/s41598-017-16526-8 (PMC5705722; doi:10.1038/s41598-017-16526-8)
Supplement: Supplementary file 1 — Supplemental Figures S1-S3 [file 41598_2017_16526_MOESM1_ESM.pdf]

Factors affecting pitch discrimination performance in a cohort of extensively phenotyped healthy volunteers

Lauren M. Smith, Alex J. Bartholomew, Lauren E. Burnham, Barbara Tillmann, Elizabeth T. Cirulli

Figure S1. Scores on short-term memory tests in those at the extreme ends of the pitch and duration discrimination distribution as compared to the rest of the population. A) Immediate story recall, pitch discrimination. B) Immediate story recall, duration discrimination. C) Digit span forward, pitch discrimination. D) Digit span forward, duration discrimination.

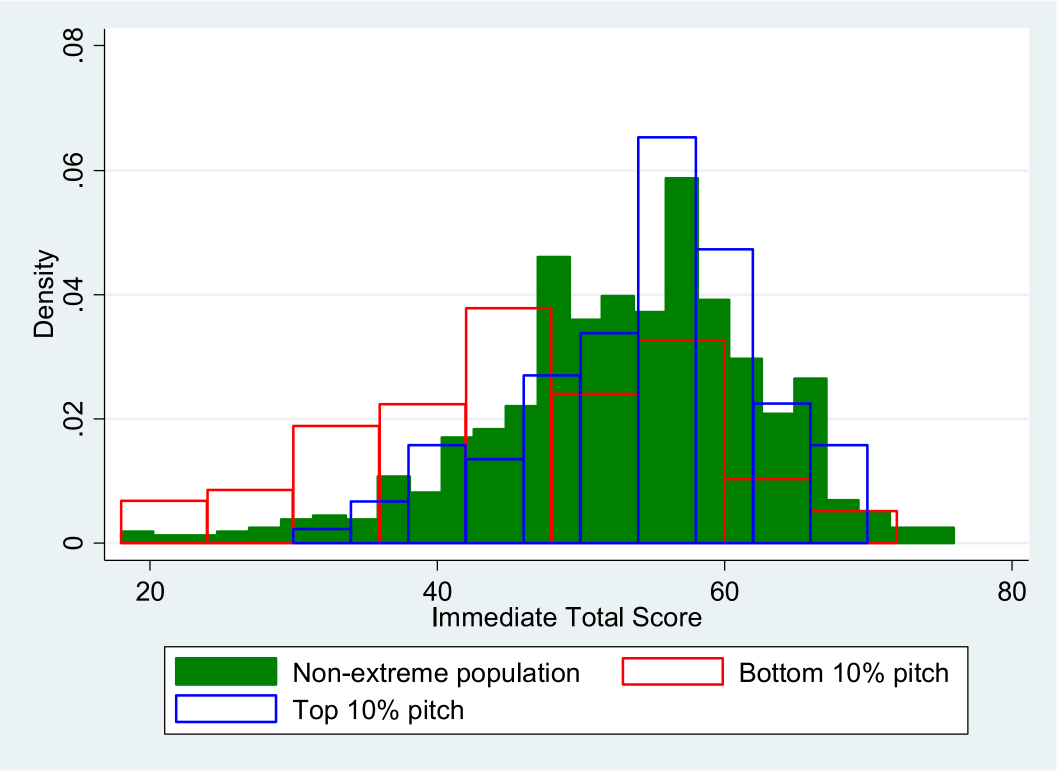

A)

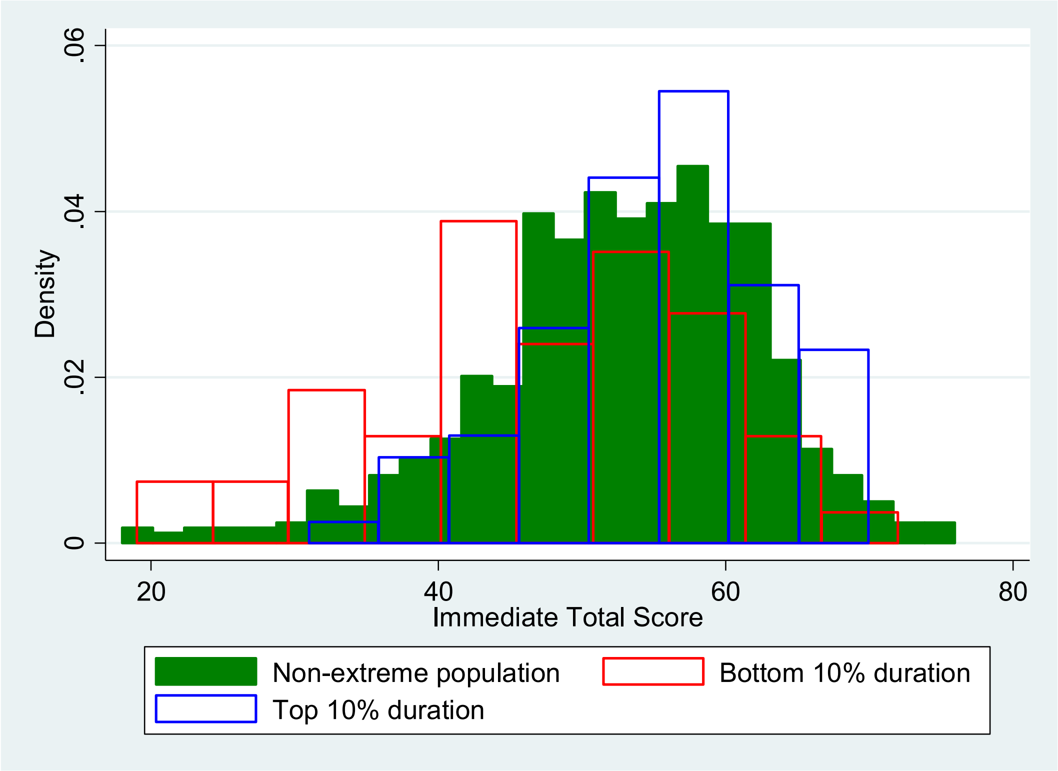

.06
.04
.02
0
Density
20
40
60
80
Immediate Total Score
Non-extreme population
Bottom 10% duration
Top 10% duration

B)

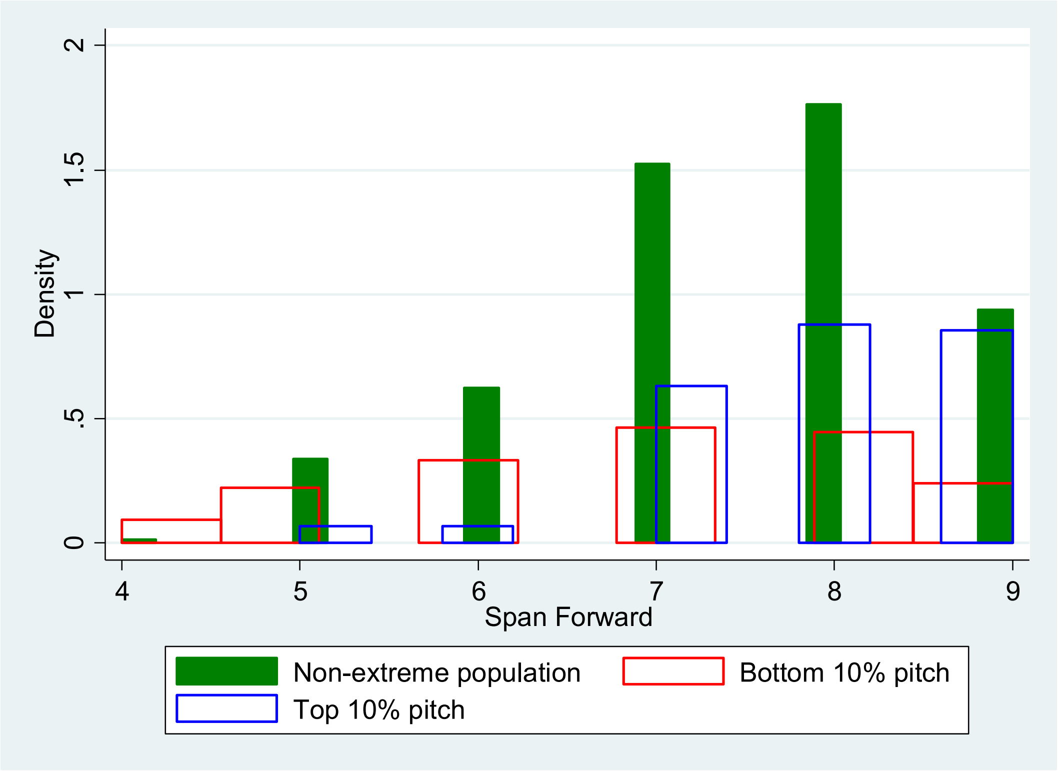

2
1.5
1
.5
0
Density
4
5
6
7
8
9
Span Forward
Non-extreme population
Bottom 10% pitch
Top 10% pitch

C)

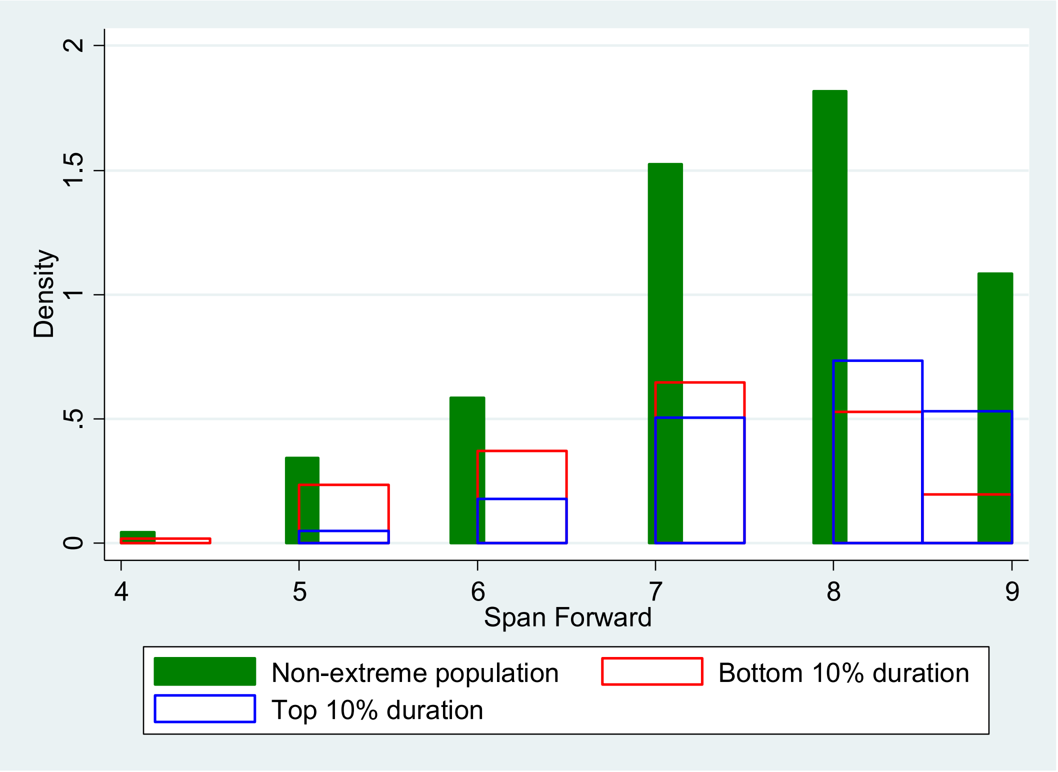

Density
0
.5
1
1.5
2
4
5
6
7
8
9
Span Forward
Non-extreme population
Bottom 10% duration
Top 10% duration

D)

Figure S2. Frequency discrimination threshold by musical training. Frequency discrimination threshold is calculated as threshold/1000, the baseline pitch. The value labeled 0.01 corresponds to a threshold of 1%, or 10 Hz. The y axis is on a log scale. A) Discrimination threshold shown according to musical training after age 6 (median threshold=0.745%, 7.45 Hz), before age 6 (median threshold=0.648%, 6.48 Hz), or no musical training (median threshold=1.133%, 11.33 Hz). A previous study found non-musicians to have a median threshold of approximately 1% while professional musicians had a median threshold of approximately 0.1%[1]; the best threshold that any of our participants attained was 0.2%. B) Correlation between discrimination threshold and total years of musical training for all participants (r^2=0.15, p<0.001) C) and for those with musical training (r_2=0.07, p<0.001)

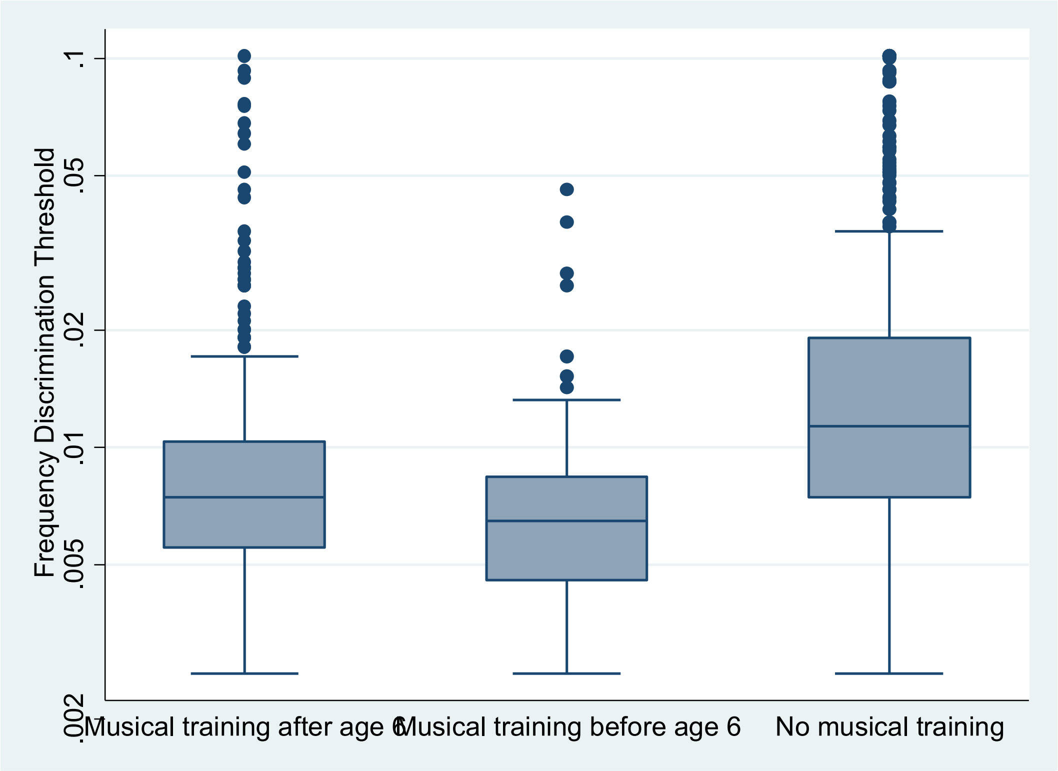

A)

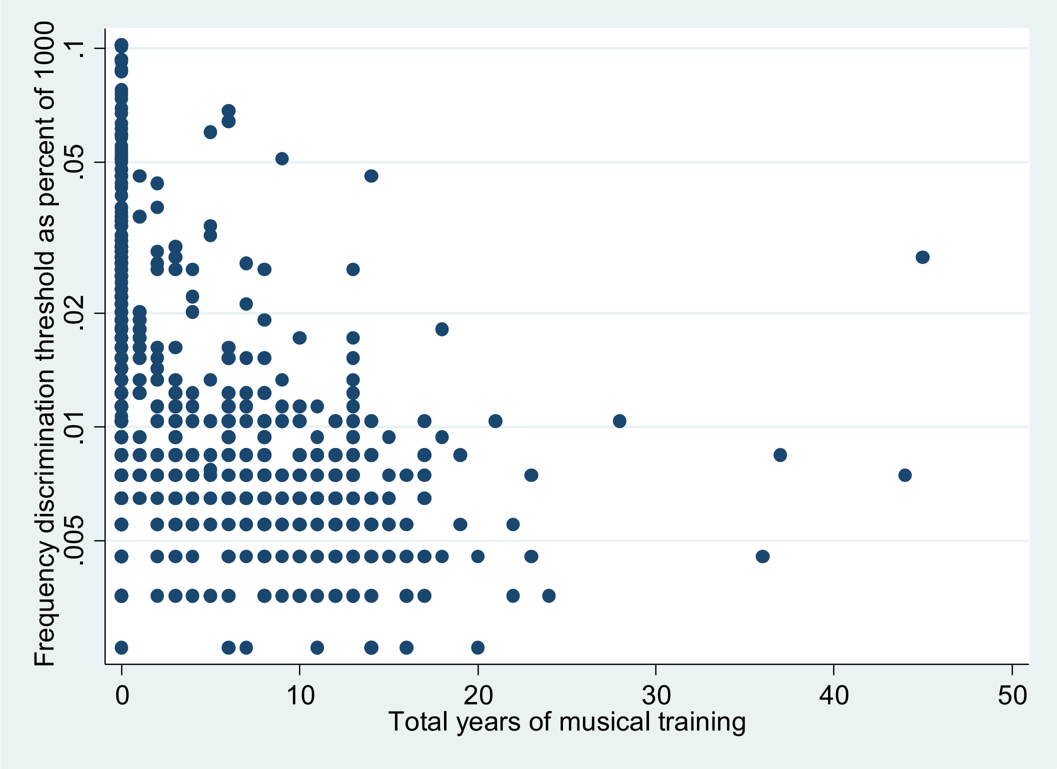

Frequency discrimination threshold as percent of 1000
.1
.05
.02
.01
.005
0
10
20
30
40
50
Total years of musical training

B)

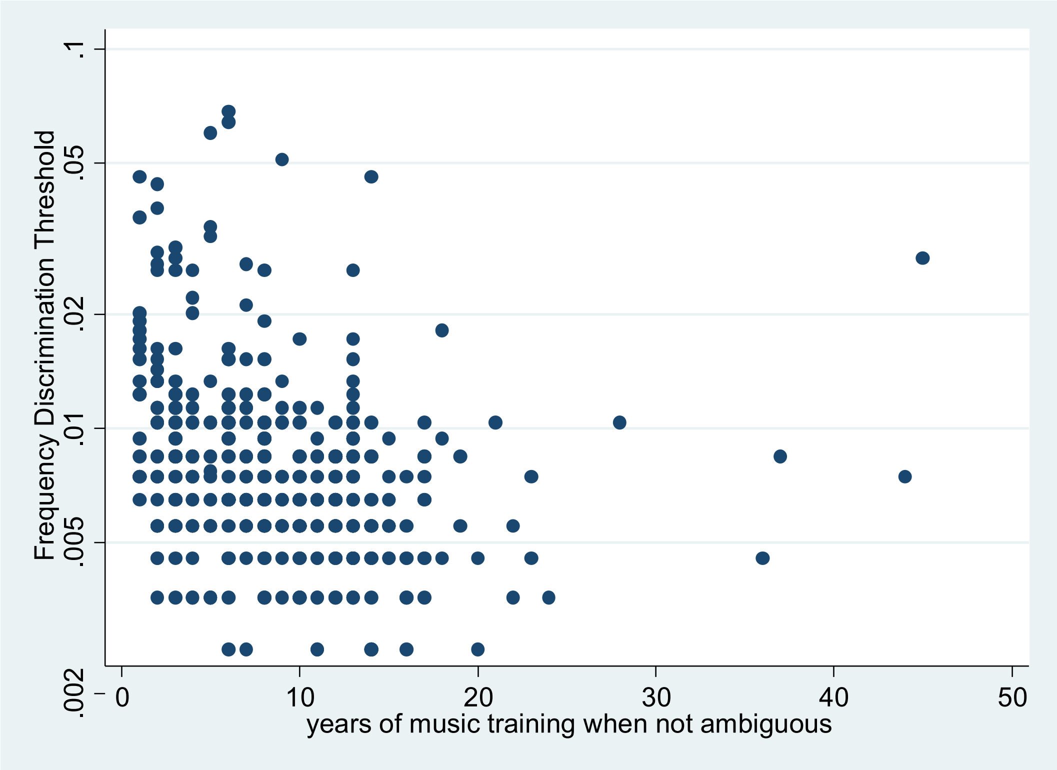

Frequency Discrimination Threshold
.1
.05
.02
.01
.005
.002
0
10
20
30
40
50
years of music training when not ambiguous

C)

Figure S3. Mean discrimination thresholds in each ethnic and musical training group. A) Pitch discrimination shown in hz on a logarithmic scale. B) Duration discrimination shown in ms. The error bars show the 95% confidence intervals.

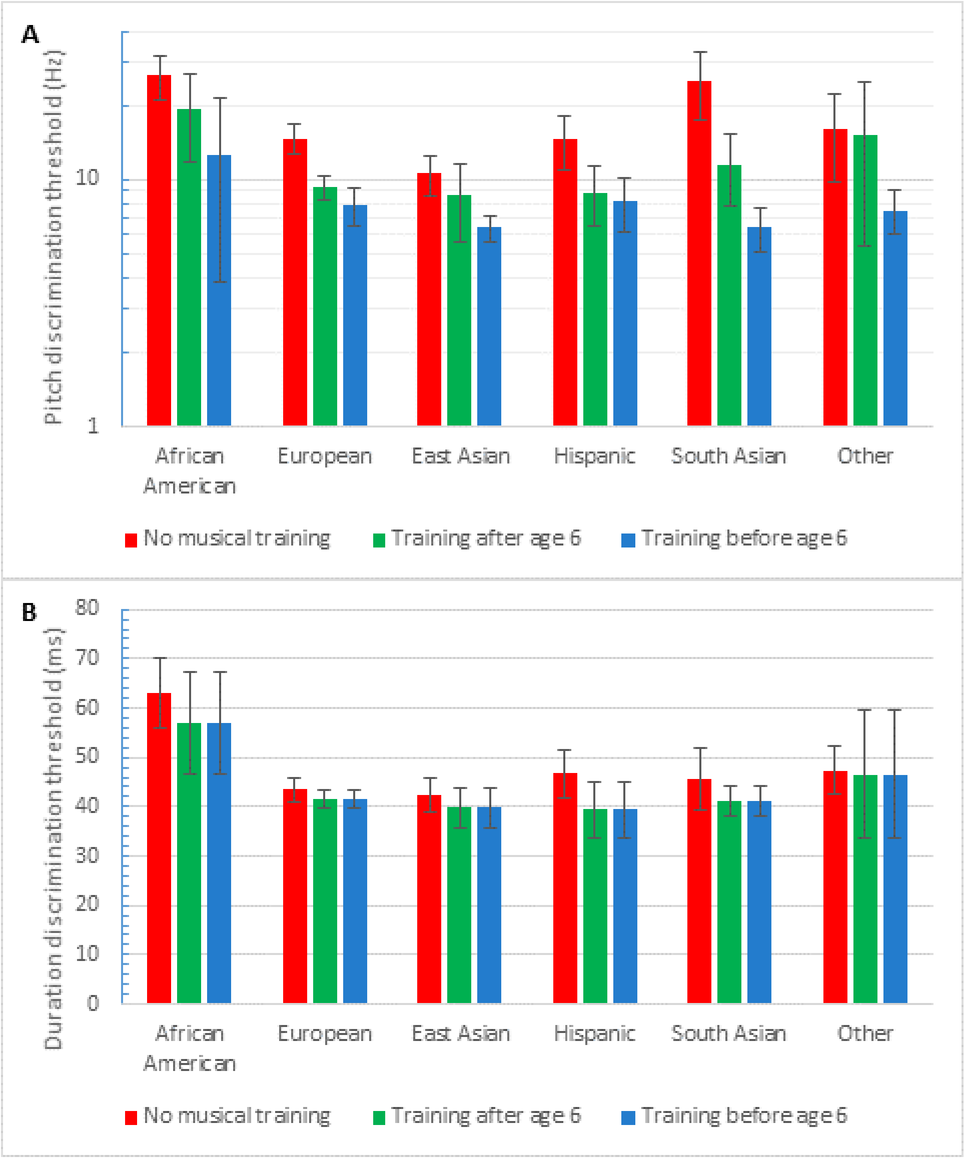

References

1. Micheyl C, Delhommeau K, Perrot X, Oxenham AJ. Influence of musical and psychoacoustical training on pitch discrimination. Hear Res. 2006;219(1-2):36-47. doi: 10.1016/j.heares.2006.05.004. PubMed PMID: 16839723.
